# Visual Preference for Biological Motion in Children and Adults with Autism Spectrum Disorder: An Eye-Tracking Study

**DOI:** 10.1007/s10803-020-04707-w

**Published:** 2020-09-20

**Authors:** Dzmitry A. Kaliukhovich, Nikolay V. Manyakov, Abigail Bangerter, Seth Ness, Andrew Skalkin, Matthew Boice, Matthew S. Goodwin, Geraldine Dawson, Robert Hendren, Bennett Leventhal, Frederick Shic, Gahan Pandina

**Affiliations:** 1 Janssen Pharmaceutica NV, Turnhoutseweg 30, 2340 Beerse, Belgium; 2 Janssen Research & Development, LLC, 1125 Trenton-Harbourton Road, Titusville, NJ 08560 USA; 3 Present Address: DataGrok, Inc., 1800 JFK Blvd Suite 300, PMB 90078, Philadelphia, PA 19103 USA; 4 Department of Health Sciences, Bouvé College of Health Sciences, Northeastern University, 312E Robinson Hall, 360 Huntington Avenue, Boston, MA 02115 USA; 5 Duke Center for Autism and Brain Development, Duke University School of Medicine, 2608 Erwin Road, Suite 30, Durham, NC 27705 USA; 6 Present Address: Center for Child Health, Behavior and Development, Seattle Children’s Research Institute, Department of Pediatrics, University of Washington School of Medicine, 2001 8th Ave Suite #400, Seattle, WA 98121 USA; 7 Benioff Children’s Hospital, University of California, San Francisco, 401 Parnassus Ave, Langley Porter, San Francisco, CA 94143-0984 USA; 8 Yale Child Study Center, Yale University School of Medicine, New Haven, USA

**Keywords:** Autism spectrum disorder, Biological motion, Biomarkers, Eye-tracking

## Abstract

Participants with autism spectrum disorder (ASD) (n = 121, mean [SD] age: 14.6 [8.0] years) and typically developing (TD) controls (n = 40, 16.4 [13.3] years) were presented with a series of videos representing biological motion on one side of a computer monitor screen and non-biological motion on the other, while their eye movements were recorded. As predicted, participants with ASD spent less overall time looking at presented stimuli than TD participants (*P* < 10^–3^) and showed less preference for biological motion (*P* < 10^–5^). Participants with ASD also had greater average latencies than TD participants of the first fixation on both biological (*P* < 0.01) and non-biological motion (*P* < 0.02). Findings suggest that individuals with ASD differ from TD individuals on multiple properties of eye movements and biological motion preference.

**Electronic supplementary material:**

The online version of this article (10.1007/s10803-020-04707-w) contains supplementary material, which is available to authorized users.

Infants typically show an early visual preference for the movement of other human beings (Blake and Shiffrar 2007; Simion et al. 2008). This attention and orientation to biological motion may be heritable, and could be a precursor to development of socio-cognitive abilities (Wang et al. 2018). Preference for biological motion may not be present to the same extent in autism spectrum disorder (ASD) (Klin et al. 2009; Pierce et al. 2011). Lack of attention to social information, in particular biological motion, may be one of the mechanisms leading to the social deficits that are characteristic of ASD (Chevallier et al. 2012; Franchini et al. 2016).

Eye-tracking (ET) measures are commonly used to determine focus of attention in passive viewing tasks. Reviews of studies of social attention in ASD have found that attention to social information is the largest discriminator between ASD and typically developing (TD) groups (2016; Frazier et al. 2017), and therefore has potential as a diagnostic biomarker, and may possibly be used as an early and independent measure of symptom severity or change in clinical trials (Dawson et al. 2012; Murias et al. 2018). At a low level, biological motion can be represented by simple point-light displays (PLD), which show movement of major joints and no other visible features (Johansson 1973; Johnson 2006). The majority of studies testing biological motion orientation and deficits in ASD make use of PLD.

Preferential viewing tasks show PLD figures that are in engaged in repetitive human activity, presented alongside scrambled dots, inverted figures, or rotating dots. Several studies found that young children with ASD, unlike TD individuals, do not preferentially attend to PLD biological motion (Annaz et al. 2012; Klin et al. 2009; Wang et al. 2015). Other studies fail to replicate between-group differences, and in some cases report a stronger preference for biological motion in preschool children with ASD (Fujisawa et al. 2014). Very few studies report on attention to PLD biological motion in older individuals with ASD. Between-group differences in viewing time of biological motion were not observed in these limited reports of adolescents and adults with ASD, leading to the supposition that preference might become less prominent with age (Fujioka et al. 2016). Other studies have found that reduced visual sensitivity to biological motion is maintained into adulthood indicating a continuation of deficits, not necessarily detected by visual preference tasks for socially relevant information (Kaiser et al. 2010). There are also indications that intelligence quotient (IQ) can impact perception of biological motion and may moderate responses in ASD (Rutherford and Troje 2012).

Beyond considering attention to biological motion as a discriminator between ASD and TD groups, biological motion has potential as an indicator, or experimental biomarker, for change in response to intervention in ASD. There is some evidence that attention to PLD biological motion may be sensitive to changes in social cognition; for example, TD adults administered oxytocin showed increased perception of PLD biological motion (Kéri and Benedek 2009). These attention changes detected by ET may be linked to neurophysiology and modulation of an electroencephalogram, wherein enhanced suppression in mu and beta bands are associated with biological motion and social stimuli perception (Perry et al. 2010). Interestingly, neural responses to biological motion are found to vary with autistic-like traits in TD adults (Puglia and Morris 2017). Moreover, in an ASD group of 19 high-functioning males without intellectual disability, a significant increase was found in first orientation to biological motion, but not latency or time spent looking, in a group administered a vasopressin 1a receptor antagonist compared to placebo (Umbricht et al. 2017).

In addition to differences in preference for biological motion, it is possible that non-social components of attention unrelated to social motivation may contribute to differences observed in passive viewing tasks in ASD. For example, overall attention to stimuli, irrespective of content, has been found to be lower in individuals with ASD compared to TD individuals (Campbell et al. 2014; Chawarska et al. 2012, 2016, 2014; Shic et al. 2011). Further, individuals with ASD compared to TD individuals may process images in different ways, and thus characteristics such as number of shifts and latency of shifts in attention may differ (Keehn et al. 2013; Landry and Bryson 2004; Sacrey et al. 2014; Sasson et al. 2008, 2011). Differences in viewing patterns, e.g., longer fixation times, relate to symptoms such as repetitive behaviors in ASD (Manyakov et al. 2018).

ET studies of social and non-social attention in ASD vary in terms of age of participants, with most studies focusing on younger children and infants and much fewer on adults (Guillon et al. 2014). Findings are rarely replicated, and magnitude of effects differs widely (Frazier et al. 2017). Research in ASD is currently focused on identification of biomarkers that may be objective, cost effective and viable diagnostic or change measures. These biomarkers may serve as a proxy for social functioning and could prove useful as endpoints in clinical trials. In order to identify such biomarkers, it is important to have both a replication of previous findings (Bradshaw et al. 2019; Klin 2018; McPartland 2017; Murias et al. 2018) and an understanding of the developmental trajectory of differences in allocation of social attention between ASD and TD groups.

## Methods

### Participants

Participants aged ≥ 6 years with a confirmed diagnosis of ASD based on clinical examination, caregiver interview and use of the Autism Diagnostic Observation Schedule, 2nd edition (ADOS-2) (Lord et al. 2012) were enrolled. Key exclusion criteria were a measured composite score on the Kaufmann Brief Intelligence Test-2 (KBIT-2) (Kaufman and Kaufman 2004) of < 60, and history of or current significant medical illness. Each site also enrolled a control sample of TD participants, aged ≥ 6 years, with a score in the normal range on the Social Communication Questionnaire (Rutter et al. 2003) who did not meet criteria for any major mental health disorder (American Psychiatric Association 2013) assessed using the Mini-International Neuropsychiatric Interview (MINI) (Hergueta et al. 1998) for those 18 years old and above, and MINI-KID caregiver interview (Sheehan et al. 2010) for participants under 18 years. There were no exclusion criteria based specifically on vision. If participants required corrective lenses to view the screen and were able to obtain calibration on the measure at the start of each test set, then their eye-tracking data were included in the study.

In total, 136 individuals with ASD and 41 TD controls completed the study. After exclusions due to technical or calibration failures, the study population included 121 (89.0%) individuals with ASD and 40 (97.6%) TD controls. Table 1 lists characteristics of each analyzed group of participants. Further details on the participant characteristics can be found in Ness et al. (2019). Note that no socioeconomic data on the participants were collected.

**Table 1:** Participant characteristics

Characteristic	Group	
	ASD	TD
	(n = 121)	(n = 40)
*Gender*, n (% *in group*)		
Male	92 (76.0)	26 (65.0)
Female	29 (24.0)	14 (35.0)
*P*-value	0.16	
*Age*, *years*		
Mean (SD)	14.6 (8.0)	16.4 (13.3)
Median (range)	12.0 (6–54)	11.5 (6–63)
*P*-value	0.69	
ADOS-2 total score, mean (SD, range)	7.6 (1.7, 4–10)	–
KBIT-2 IQ composite score, mean (SD, range)	98.5 (20.0, 60–136)	–

The two groups are matched in terms of gender (χ^2^ test) and age (two-sample Kolmogorov–Smirnov test). n indicates the number of participants

*ADOS-2* Autism Diagnostic Observation Schedule, 2nd edition, *KBIT-2* Kaufmann Brief Intelligence Test-2, *SD* standard deviation

### Biological Motion

The biological motion task consisted of videos in which two PLD animations were shown side-by-side. A PLD representing biological motion was shown on one side and a non-biological PLD was shown on the other. These stimuli were previously used by and reported by Umbricht et al. (2017). The PLDs were made up of dynamic black-point displays on a grey background (Supplementary Fig. 1). Each video lasted approximately four seconds. The biological/non-biological side was counterbalanced across presentations. Animations of biological motion were derived from a human actor’s performance and retrieved from the Carnegie Mellon University (CMU) Motion Capture Database (CMU Graphics Lab 2011). In contrast, animations of non-biological motion were computer-generated animations of moving dots, either phase-scrambled versions of the biological motion or continuous rotation (i.e. spinning around the vertical axis) of the first frame of the biological motion animation (see Supplementary Material for the analysis of attention to the two types of non-biological motion stimuli). All stimuli contained the same number of dots.

Biological and non-biological motion stimuli were matched in terms of motion complexity and speed of movement (see also Supplementary Material). There was an equal amount of novel stimuli in both the biological and non-biological motion condition. Both phase-scrambled and rotational control conditions were created using an original biological motion stimulus as a template. Phase-scrambled motion was created by time shifting each biological-motion point’s motion by a random time offset. These time offsets were randomly selected to sample uniformly between − 417 and 417 ms based on the number of points shown. The periodicity of this offset (833 ms) was ascertained by examining the dominant period of biological motion movement across clips (approximately 100 frames at 120 frames per second) via autocorrelation. This process guaranteed the total relative local motion of individual point-lights was comparable between phase-scrambled and unscrambled biological motion stimuli. The rotational point-light display was created by computing the dominant periodicity of hip movement in the source biological motion stimuli via autocorrelation and then rotating the first frame of the biological motion stimulus about the z-axis (following the spine in upright walking) at a constant speed given by hip periodicity. Stimuli were designed so that the first frame shown would be comparable across all conditions (unscrambled biological motion, scrambled biological motion, rotation).

This biological motion task was part of a large, observational, multi-center study conducted from 06 July 2015 to 14 October 2016 at nine study sites in the US (ClinicalTrials.gov, NCT02668991) and consisted of passive viewing tasks (Bangerter et al. 2020a, 2020b; Jagannatha et al. 2019; Manfredonia et al. 2018; Manyakov et al. 2018; Ness et al. 2019; Sargsyan et al. 2019). In this study, both groups of participants completed the same set of biosensor tasks. The total viewing time was approximately 40 min, including videos and other static stimuli presentations, divided into three sets, between which participants were allowed to take a break. Sixty different videos of biological motion were interspersed between these other stimuli and presented in 6 blocks of 10 videos (2 blocks within each set). An inter-stimulus slide that contained a cartoon image in the center of a grey screen was used for a duration of 1.5–2 s to re-orientate participants towards the center of the screen before each video, though no control was performed to verify the latter.

### Behavior Rating Scales

Scale data were collected concurrently with eye-tracking data. Parents or caregivers of individuals with ASD were required to spend at least 3 days per week with participants. They completed the following scales:

- Autism Behavior Inventory (ABI), a rating scale developed to assess change in core and associated symptoms of autism (Bangerter et al. 2017).
- Aberrant Behavior Checklist—Community (ABC) assesses general behaviors (Aman et al. 2004; Aman and Singh 2017).
- Child Adolescent Symptom Inventory-Anxiety (CASI-Anx) is a 20-item subset of the CASI which assesses anxiety (Gadow and Sprafkin 1997).
- Social Responsiveness Scale 2™ (SRS-2) identifies presence and severity of social impairment due to ASD (Constantino et al. 2003).
- Repetitive Behavior Scale—Revised (RBS-R) provides a quantitative measure of the full spectrum of repetitive behaviors (Bodfish et al. 1999).

Each of the above scales and ADOS-2 consist of several subscales that reflect different ASD symptoms. All subscales used in this study are presented in Table 2. Standard scores for the above scales and the calibrated severity score of the ADOS-2 were used in the reported analyses.

**Table 2:** Preference for biological motion in the ASD participants across different levels of symptom severity

	Symptom severity level		
	Mild	Moderate	Severe
*ABI*	n = 39	n = 39	n = 39
Core ASD symptom scale score	**53.7 (0.02)**	**54.8 (< 10**^**–4**^**)**	**54.2 (0.01)**
Challenging behavior	52.4 (0.12)	**55.1 (< 10**^**–3**^**)**	**55.4 (< 10**^**–4**^**)**
Mental health	52.3 (0.06)	**54.4 (< 10**^**–3**^**)**	**56.1 (< 10**^**–4**^**)**
Restrictive repetitive behaviors	**52.9 (0.05)**	**53.7 (0.01)**	**56.2 (< 10**^**–4**^**)**
Self-regulation	52.5 (0.15)	**54.4 (< 10**^**–3**^**)**	**55.8 (< 10**^**–4**^**)**
Social communication	**54.6 (0.01)**	**54.5 (< 10**^**–3**^**)**	**53.6 (0.01)**
*ADOS-2*	n = 40	n = 40	n = 41
Restricted and repetitive behavior	**53.8 (0.01)**	**54.4 (0.01)**	**55.2 (< 10**^**–4**^**)**
Social affect	**54.9 (0.01)**	**55.2 (< 10**^**–4**^**)**	**53.3 (0.01)**
Total score	**56.0 (< 10**^**–4**^**)**	**53.2 (0.05)**	**54.2 (< 10**^**–3**^**)**
*ABC*	n = 40	n = 40	n = 41
Hyperactivity non-compliance	**53.2 (0.02)**	**54.7 (0.01)**	**55.5 (< 10**^**–4**^**)**
Inappropriate speech	**53.3 (0.05)**	**53.9 (< 10**^**–3**^**)**	**56.1 (< 10**^**–4**^**)**
Irritability	51.7 (0.08)	**56.2 (< 10**^**–4**^**)**	**55.4 (< 10**^**–4**^**)**
Lethargy social withdrawal	**55.8 (< 10**^**–5**^**)**	**53.6 (0.02)**	**54.0 (0.01)**
Stereotypic behavior	**53.6 (0.03)**	**53.6 (0.01)**	**56.2 (< 10**^**–5**^**)**
*CASI-Anx*	n = 40	n = 40	n = 41
Total score	**54.9 (0.01)**	**54.2 (0.01)**	**54.4 (< 10**^**–3**^**)**
*RBS-R*	n = 40	n = 40	n = 41
Compulsive behavior	**53.0 (0.03)**	**55.7 (< 10**^**–3**^**)**	**54.7 (< 10**^**–3**^**)**
Ritualistic behavior	52.5 (0.11)	**54.4 (< 10**^**–3**^**)**	**56.4 (< 10**^**–5**^**)**
Restricted behavior	**53.7 (0.01)**	**53.8 (0.01)**	**55.9 (< 10**^**–4**^**)**
Sameness behavior	52.8 (0.08)	**54.5 (< 10**^**–3**^**)**	**56.1 (< 10**^**–4**^**)**
Self-injurious behavior	**53.1 (0.02)**	**54.2 (0.02)**	**56.1 (< 10**^**–6**^**)**
Stereotyped behavior	**54.5 (0.01)**	**52.8 (0.02)**	**56.1 (< 10**^**–5**^**)**
Total score	52.4 (0.15)	**54.5 (< 10**^**–3**^**)**	**56.5 (< 10**^**–5**^**)**
*SRS-2*	n = 40	n = 40	n = 40
Social awareness	**54.2 (< 10**^**–3**^**)**	**53.8 (0.01)**	**55.1 (< 10**^**–3**^**)**
Social cognition	**53.2 (0.01)**	**53.8 (0.01)**	**56.2 (< 10**^**–4**^**)**
Social communication	**54.0 (0.01)**	**54.4 (< 10**^**–3**^**)**	**54.7 (< 10**^**–3**^**)**
Social motivation	**55.2 (< 10**^**–3**^**)**	**53.6 (0.01)**	**54.3 (< 10**^**–3**^**)**
Restricted interests and repetitive behavior	**52.5 (0.04)**	**54.7 (< 10**^**–3**^**)**	**55.9 (< 10**^**–4**^**)**
Social communication and interaction	**53.1 (0.01)**	**54.6 (0.01)**	**55.4 (< 10**^**–4**^**)**
Total score	**53.0 (0.02)**	**54.3 (0.01)**	**55.8 (< 10**^**–4**^**)**

Mean preference for biological motion is computed for each symptom and severity level separately (Supplementary Table 4), with n indicating the number of participants entering the computation. *P*-values in parentheses indicate a significant difference from the chance level (50%, i.e. equal fixation on both types of stimuli), with the values below 0.05 being highlighted in bold

*ABC* Aberrant Behavior Checklist—Community, *ABI* Autism Behavior Inventory, *ADOS-2* Autism Diagnostic Observation Schedule, 2nd edition, *ASD* autism spectrum disorder, *CASI-Anx* Child Adolescent Symptom Inventory—Anxiety, *RBS-R* Repetitive Behavior Scale—Revised, *SRS-2* Social Responsiveness Scale 2™, *TD* typically developing

### Procedure

Participants sat in a comfortable chair approximately 60 cm from a 23-inch computer screen (1920 × 1080 pixels). The height of the chair and screen were adjusted to ensure that participants’ eyes were level with the center of the screen. ET data were collected using the Tobii X2 eye tracker, with a sampling rate of 30 Hz, mounted below the screen. iMotions Biometric Research Platform (https://imotions.com/) was used for stimuli presentation, data synchronization, and automatic calibration. Participants were allowed to freely observe presented stimuli. To ensure a high accuracy of the eye movement recordings (e.g., Blignaut and Wium 2014), before each experimental set a five-point calibration procedure consisting of animated cartoon characters paired with an auditory cue was performed. The calibration procedure was aimed to reach the mean distance between the participant’s gaze direction and the target points of less than 0.5° of visual angle.

### Data Analysis

For each frame, circles with the radius of approximately 3.7° of visual angle centered at each “black dot” corresponding to (non-)biological motion were drawn. Combination of such circles that corresponded to dots representing biological motion determined a biological motion region of interest (ROI). Similarly, a non-biological motion ROI was determined. Supplementary Fig. 1 shows two dynamic ROIs on a single example frame. Fixations were identified using the Binocular-Individual Threshold algorithm (van der Lans et al. 2011). The minimum required number of tracked samples and the maximum acceptable number of consecutive untracked samples in the eye tracking signal in a single fixation were both set to three. The following eye movement metrics were computed:

- Total valid time (%)—percentage of time participant gazed at the screen, which is the opposite to data loss.
- Preference for biological motion (%)—ratio of total time a participant spent looking at biological motion to total time he/she spent looking at biological and non-biological motion together.
- Percentage of time the first fixation was on biological motion (%)—percentage of time participant first fixated on biological rather than on non-biological motion.
- Average latency of the first fixation on biological (non-biological) motion (msec)—averaged (across all videos) time from stimulus onset until participant fixated for the first time on a ROI defining biological (non-biological) motion. Note that no control was performed to verify participant’s attention on the center of the screen in the beginning of each video stimulus presentation.

Analysis of covariance (ANCOVA) was conducted to compare eye movement metrics between the ASD and TD groups. Specifically, a single eye movement metric was used as a dependent variable, whereas participant group served as an independent variable. Participant’s age and gender entered the analysis as additional factors. Note that the KBIT-2 test was only administered in the ASD group and, thus, participant’s IQ score could not be included in the analysis as an additional factor. Supplementary Tables 6 and 7 show the results of tests for the homogeneity of variance assumption made in the ANCOVA models, and the results obtained with the same ANCOVA models as correcting for the violations of the assumption, respectively. Since ANCOVA models including two-factor interactions between participant group and the two other covariates did not significantly differ from those without any interaction (analysis of variance: all *P*-values > 0.18), statistical inference was based on the models without interactions. Note also that none of the scale data (see *Behavior Rating Scales*) entered any of the ANCOVA models. To test for effect of outliers on the obtained results, the same analyses were repeated after removing potential outliers from the analyzed dataset (Supplementary Table 1). To test for effect of site on the eye movement metrics, for each metric we conducted ANCOVA similar to that described above but additionally included a site identifier as an independent categorical variable. Similarly, to test for an interaction between participant’s age and group, the original ANCOVA models included an interaction term between these two factors. To test for effect of IQ on the differences in eye movement metrics between the two groups of participants, the entire ASD sample was divided into three separate groups based on the level of IQ. The tested levels represented the ASD participants with a low (range of the KBIT-2 IQ composite score: 60–84; n = 34 participants), average/normal (85–115; n = 63), and high (116–136; n = 24) IQ. The same ANCOVA’s as above were conducted combining the data of all TD participants and individuals with ASD with a specific level of IQ (Supplementary Table 2). The effect size of participant group on each eye movement metric was estimated using Cohen’s d and partial eta-squared (η_p_^2^).

The Wilcoxon signed rank test was applied to compare preference for biological motion and “% time the first fixation was on biological motion” against the chance level (50%) in each of the two groups of participants separately. Similarly, to compare average latency of the first fixation between biological and non-biological stimuli within a single group of participants, the Wilcoxon matched pairs signed rank test was used.

Relationships between the eye movement metrics and different ASD symptoms were assessed using Spearman partial correlations, with participant’s age, gender and KBIT-2 IQ composite score serving as covariates (Supplementary Table 5). Spearman correlations were also used to assess relationships between the eye movement metrics and KBIT-2 IQ composite score (Supplementary Table 3) as well as between preference for biological motion and participant’s age (Supplementary Fig. 2). All reported correlations (r_S_) were computed using the data of all ASD participants. The choice of Spearman correlations was attributed to a generally lower susceptibility of this type of correlations to potential outliers present in the data, as compared to Pearson correlations. Note, however, that qualitatively similar results were also observed when using Pearson correlations.

All statistical analyses were performed in MATLAB and R, with package “ppcor” in R used to compute partial correlations (Kim 2015). All reported *P*-values were two-sided, except those generated by ANCOVAs and based on F-statistic. The *P*-values corresponding to the differences in eye movement metrics between the two groups of participants were adjusted for multiple comparisons (five tests) using the Benjamini–Hochberg procedure (false discovery rate = 5%) and are reported as such throughout the main text. Non-adjusted, original *P*-values of all ANCOVA’s are listed in Supplementary Tables 1 and 2. No other correction for multiple comparisons was performed, since the study was exploratory rather than hypothesis-driven. Note that the number of statistical comparisons and, thus, the exact cut-off for significant *P*-values in each analysis was debatable. For example, the total number of tests entering the only reported adjustment for multiple comparisons (see above) was set to five. Alternatively, it could have been set to 15 when additionally counting the tests run to assess the effect of participant’s gender and age on the observed results. Adjustment for multiple comparisons for the relationships between the eye movement metrics and ASD symptoms (Supplementary Table 5) could have been done in multiple ways: for each symptom and behavior rating scale separately but across all five eye movement metrics, for each eye movement metric separately but across all symptoms and behavior rating scales, and combining all tests regardless eye movement metric, symptom and behavior rating scale. For the reasons outlined above, the *P*-values were reported “as is” with values < 0.05 considered significant.

## Results

### Comparison of Eye Movement Metrics Between ASD and TD Participants

**Fig. 1:**
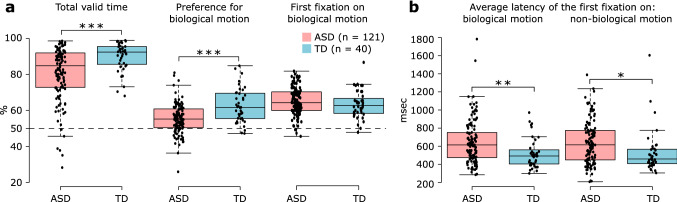
Differences in the eye movement metrics between ASD and TD participants

Differences in eye movement metrics between ASD (n = 121 participants) and TD (n = 40) groups are shown in Fig. 1. Analysis of covariance revealed a significant effect of participant group on all metrics (all *P*-values corrected for multiple comparisons < 0.03), except “% time the first fixation was on biological motion” (Supplementary Table 1). On average, the ASD group showed a significantly lower level of visual attention to presented stimuli than the TD group (% total valid time; ASD vs. TD, mean: 80.0% vs. 89.2%; Cohen’s d = 0.68, η_p_^2^ = 0.08, *P* < 10^–3^). In comparison to the TD group, the ASD group spent significantly less time looking at stimuli that depicted biological as opposed to non-biological motion (% preference for biological motion; ASD vs. TD, mean: 54.5% vs. 61.9%; Cohen’s d = 0.87, η_p_^2^ = 0.14, *P* < 10^–5^). Overall preference for biological motion was significantly greater than the chance level (50%, equal looking time for both types of stimuli) in both groups of participants (both *P*-values < 10^–8^). Preference for biological motion was the only eye movement metric that was significantly related to age, with older individuals (ASD and TD) showing a decrease in preference with age (Supplementary Table 1) (Supplementary Fig. 2). Percentage of time the first fixation was on biological motion was similar between the two groups of participants (ASD vs. TD, mean: 54.2% vs. 52.7%; Cohen’s d = 0.25, η_p_^2^ = 0.01, *P* = 0.26). In both groups of participants, percentage of time the first fixation was on biological motion significantly exceeded the chance level (in both groups *P*-value < 0.01). Individuals with ASD showed greater average latencies of the first fixation on both biological (ASD vs. TD, mean: 649.1 ms vs. 521.3 ms; Cohen’s d = 0.56, η_p_^2^ = 0.05, *P* < 0.01) and non-biological motion (639.7 ms vs. 530.9 ms; Cohen’s d = 0.46, η_p_^2^ = 0.04, *P* < 0.03), as compared to TD controls. There was no significant difference in average latency of the first fixation between biological and non-biological stimuli when data from each group were analyzed separately (both *P*-values > 0.94). Qualitatively similar results were observed when accounting for an interaction between participant’s age and group (the interaction term in ANCOVA’s: all five *P*-values > 0.24), a potential effect of site on the eye movement metrics (the effect of site in ANCOVA’s: all five *P*-values > 0.10), and the violations of the homogeneity of variance assumption in the analyses (Supplementary Tables 6 and 7). Furthermore, the effect of participant group on all five metrics persisted after removing potential outliers present in the data (Supplementary Table 1).

Restricting analyses to the groups of individuals with ASD with a specific level of IQ (low: n = 34 participants; average/normal: n = 63; high: n = 24) showed qualitatively similar results (Supplementary Table 2). Interestingly, as the IQ of the ASD group increased, “% total valid time” and average latency of the first fixation on either type of stimuli were more similar to those of the TD group. On the contrary, as the IQ of the ASD group increased, preference for biological motion deviated more from that observed in the TD group. Importantly, no systematic relationship between any of the five eye movement metrics and KBIT-2 IQ composite score was observed across the three tested IQ levels (Supplementary Table 3). Altogether, this rules out an explanation of the observed differences in the eye movement metrics between the two groups of participants by IQ.

### Preference for Biological Motion Across Different Levels of ASD Symptom Severity

To test for effect of severity of ASD symptoms on preference for biological motion for each behavior rating scale and symptom, participants with ASD were ranked according to the numeric score of that symptom and then split into three similarly-sized, non-overlapping groups (mild, moderate, severe) (Supplementary Table 4). For all five caregiver-reported behavior rating scales and ADOS-2, all symptoms, and three severity groupings, mean preference for biological motion exceeded the chance level (50%), reaching statistical significance in 80 out of 87 (92%) comparisons (all *P*-values < 0.05) (Table 2). All non-significant comparisons were in the mild symptom severity category.

### Relationship Between Eye Movement Metrics and ASD Symptoms

To assess relationships between eye movement metrics and different ASD symptoms, as assessed by five caregiver-reported behavior rating scales and ADOS-2, each metric (n = 5) was examined for correlation with each symptom numeric score (n = 29). Only 5 of 145 computed correlation coefficients (3.4%) reached statistical significance (Supplementary Table 5; Supplementary Figs. 4–8). The ADOS-2 “restricted and repetitive behavior” significantly and positively correlated with % total valid time (r_S_ = 0.192, *P* < 0.04). Similarly, preference for biological motion correlated with the ABI “mental health” (r_S_ = 0.185, *P* < 0.05). Lastly, average latency of the first fixation on non-biological motion significantly and negatively correlated with the ABC “stereotypic behavior” (r_S_ = − 0.245, *P* < 0.01), the RBS-R “restricted behavior” (r_S_ = − 0.192, *P* < 0.04) and “stereotyped behavior” (r_S_ = -− 0.191, *P* < 0.04).

## Discussion

We report differences in eye movements between individuals with ASD and TD controls while viewing biological motion. Overall, individuals with ASD paid less attention to the presented stimuli than TD controls. In comparison to TD controls, individuals with ASD spent less time looking at biological vs. non-biological motion, accounting for differences in total attention to stimuli. Nevertheless, individuals with ASD revealed a subtle preference for biological motion. In addition, both the ASD and TD groups showed a subtle preference to make their first fixation on biological motion, and this preference did not differ between the two groups. Moreover, individuals with ASD showed a greater average latency of the first fixation than TD controls regardless the type of presented stimuli.

Our results suggest that the reduced preference for biological motion observed in studies of younger children (Annaz et al. 2012; Klin et al. 2009; Wang et al. 2015) remain in older individuals with ASD. Differences between the ASD and TD groups in time spent looking at biological motion were persistent across phenotypic groups of varying severity as determined by behavior rating scales, suggesting that reduced preferences for biological motion were evident in individuals with ASD regardless of severity. Although the preference for biological motion over non-biological motion in individuals with ASD was less than in the TD group, our results consistently showed that across phenotypic groups of individuals with ASD this preference was still significantly greater than expected by chance. Individuals with ASD are still more likely to look at biological motion than non-biological motion, but less likely than the TD group. Interestingly, familiarization with the stimuli that could have been presumably built-up across stimulus presentations did not modulate preference for biological motion in either group of participants (see Supplementary Material). Importantly, effect sizes for preferences were small, which may explain the lack of consistency in previously reported findings, particularly in studies with small sample sizes.

Sifre et al. (2018) observed a U-shaped curve in the occurrence of biological motion preference in typically developing infants from neonates to 2 years, with no evidence of the preference seen in 2-month olds. They suggest that this observation is the result of a shift from experience-expectant to experience-dependent mechanisms that drive visual attention to biological motion, occurring at around 2 months of age. Our observation that, despite an overall preference for biological motion, a reduced preference in comparison to the TD group remains in older children and adults with ASD, which may be accounted for by differences in experience-dependent mechanisms in this group. For example, the Social Motivation Theory (Chevallier et al. 2012) suggests that less reward value is assigned to social stimuli, and this can be linked to motivation to engage socially and increased social impairments in ASD (Dawson et al. 2004). Reward value of biological motion specifically was established in a group of TD individuals (Williams and Cross 2018). Individuals in this group with more autistic traits showed reduced motivation for biological motion. Although we did not find that reduced preference for biological motion was related to any caregiver reported observation of social skills in the ASD group alone, the reduced preference observed in comparison to the TD group may reflect differences in reward values associated with biological motion between the groups. If so, it is possible that interventions that impact on reward values may lead to changes in biological motion preference that precede desired improvements in social motivation. In this way, increase in biological motion preference in ASD may serve as a biomarker for change in response to intervention.

There were a few relationships between any of the eye tracking metrics in ASD and behavioral reports using the behavioral rating scales. Given the modest size of these correlations, none of the observed relationships would remain if adjustments were made for multiple comparisons. Altogether, we believe that our data do not provide compelling evidence for the existence of a relationship between severity of ASD symptoms and preference for biological motion. Instead, these correlations should be interpreted with a great caution and rather be used to inform future research about the existence of potential links between behavioral reports and eye-tracking measures. Note, however, that the lack of correlations appears to be genuine given controls for participant’s age, gender and IQ as well as the size and heterogeneity of the ASD sample used to compute the correlations. Furthermore, the significant differences observed in the eye-tracking metrics between the two groups of participants suggest that these metrics convey information on ASD that can be orthogonal to that captured by behavior rating scales. An interesting observation was the relationship with IQ, that individuals with ASD who had a higher IQ spent less time looking at biological motion. It is possible that different mechanisms are driving attention to or away from biological motion across development in ASD. Contrary to our results, Rutherford and Troje (2012) found that perception of biological motion was increased with higher IQ in ASD. It is possible that individuals with ASD and higher IQ in the current study switched attention more quickly to non-biological motion following an initial fixation and perception of biological motion, thus leading to increased time spent on viewing non-biological motion compared to those with ASD and lower IQ. The relationship between social attention and IQ in ASD warrants further research. Besides the relationship with IQ, preference for biological motion tended to slightly decrease with age across both groups of participants. Though this relationship would not survive adjustment for multiple comparisons and disappeared after removing potential outliers present in the data, it may reflect change over time in cognitive processes that impact preference for biological motion as an individual matures. However, the latter proposal is challenged by the lack of effect of age on attention to social stimuli in the eye-tracking studies of ASD, as is reported in two recent meta-analysis studies by 2016 and Frazier et al. (2017).

Although latency to the first fixation was estimated without verifying participant’s attention on the center of the screen in the beginning of each video stimulus presentation, which could have had an impact on the estimates, this should not have influenced between-group comparisons. Longer latency to first fixation was observed in the ASD group, regardless of whether first fixation was to biological or non-biological motion. This suggests that allocation of visual attention may differ in ASD, regardless of the nature of the stimuli. For example, individuals with ASD may experience difficulties with disengagement and might be impacted by the use of a cartoon image in place of the cross hair in this task. Slower first fixation may also be the result of a more cognitively-driven approach at first fixation, and/or differences in motion sensitivity/perception. The current task was not developed to study these potential differences, and since features for first fixation were estimated irrespective of whether participants were looking at the screen between clip presentations, it may be that the reduced valid time viewing the screen could account for some of the delays in first fixation in ASD. However, observed differences in latency to first fixation warrant further investigation to understand the mechanisms that might impact allocation of visual attention in ASD.

### Limitations

This study was part of a larger prospective, non-observational study designed to develop measures of change in ASD (Ness et al. 2019). One focus of the study was on obtaining quality data across a number of tasks and biosensors within the ASD population. Given the heterogeneity of this group, the aim was to maximize the collection of data from this sample. A group of TD individuals was added for comparison. As this was not the main focus of the study, this led to a smaller and less characterized TD group. In particular, the TD group did not have IQ measures, which limits inferences about the impact of IQ on performance. In addition, controls for multiple comparison were not performed in these analyses. The current results should be considered exploratory, and further validation in additional studies is required.

This study did not involve an intervention, and therefore the responsiveness to change of the features that discriminate between the ASD and TD groups could not be assessed, and this is a next step for an ongoing intervention study (NCT03664232). However, unlike Umbricht et al. (2017) who determined that first orientation to biological motion was less in an ASD group, and increased following vasopressin 1a receptor antagonist administration, we did not find evidence of between-group differences for this feature. Future studies are required to determine whether attention to biological motion can be increased in response to intervention, and whether this could be a precursor to other observable change in social interaction.

## Summary and Conclusions

The examined group of individuals with ASD, aged 6 to adult, showed differences in visual attention to biological motion compared to TD individuals. This agrees with previously reported observations in younger children with ASD. The differences observed in this older group of individuals with ASD may also reflect differences in reward allocation of social stimuli and support the Social Motivation Theory. Though the revealed differences are small, they may contribute to observable social interaction difficulties in ASD over time. Therefore, differences in allocation of visual attention may be a risk marker or useful contributor to diagnostic criteria in ASD and could leverage monitoring outcomes in clinical trials. Nevertheless, future interventional studies are still needed to determine whether these group differences are sensitive to change in individuals with ASD.

## Electronic supplementary material

Below is the link to the electronic supplementary material.

Supplementary file1 (DOCX 197 kb)

## Data Availability

The data sharing policy of Janssen Pharmaceutical Companies of Johnson & Johnson is available at https://www.janssen.com/clinical-trials/transparency. As noted on this site, requests for access to the study data can be submitted through Yale Open Data Access (YODA) Project site at https://yoda.yale.edu.
