# Supplementary material for: Visual Preference for Biological Motion in Children and Adults with Autism Spectrum Disorder: An Eye-Tracking Study
Source: J Autism Dev Disord. 2020 Sep 20;51(7):2369–80. doi: 10.1007/s10803-020-04707-w (PMC8189980; doi:10.1007/s10803-020-04707-w)
Supplement: Supplementary file 1 — Supplementary file1 (DOCX 197 kb) [file 10803_2020_4707_MOESM1_ESM.docx]

**Supplementary Material**

**Article title:** Visual preference for biological motion in children and adults with autism spectrum disorder: an eye-tracking study.

**Journal name:** Journal of Autism and Developmental Disorders.

**Author names:** Dzmitry A. Kaliukhovich, Nikolay V. Manyakov, Abigail Bangerter, Seth Ness, Andrew Skalkin, Matthew Boice, Matthew S. Goodwin, Geraldine Dawson, Robert Hendren, Bennett Leventhal, Frederick Shic, Gahan Pandina.

**Corresponding author:** Dzmitry A. Kaliukhovich, Janssen Pharmaceutica NV, Turnhoutseweg 30, 2340 Beerse, Belgium, phone: +32 479865613, e-maill: [dkaliukh@its.jnj.com](mailto:dkaliukh@its.jnj.com).

**Supplementary Material**

**Table of Contents**

**Supplementary Table 1.** Results obtained with ANCOVA on different selections of participants’ data.

**Supplementary Table 2.** Results obtained with ANCOVA using groups of the ASD participants with varying levels of intelligence quotient.

**Supplementary Table 3.** Spearman correlations between the eye movement metrics and KBIT-2 IQ composite score in individuals with ASD.

**Supplementary Table 4.** Numeric scores of different ASD symptoms used to define three severity levels in Table 2.

**Supplementary Table 5.** Spearman partial correlations between the eye movement metrics and the numeric scores of different ASD symptoms.

**Supplementary Table 6.** Comparison of variance of the eye movement metrics between the two groups of participants.

**Supplementary Table 7.** Results obtained with ANCOVA when correcting for the violations of homogeneity of variance.

**Supplementary Figure 1.** Biological (left) and non-biological (right) motions with corresponding ROIs.

**Supplementary Figure 2.** Scatter plot of preference for biological motion as a function of participant’s age.

**Supplementary Figure 3.** Histograms of average latency of the first fixation on biological motion for each participant’s gender separately.

**Supplementary Figure 4.** Scatter plot of % total valid time as a function of ASD severity level, as reflected in the ADOS “restricted and repetitive behavior” score.

**Supplementary Figure 5.** Scatter plot of preference for biological motion as a function of ASD severity level, as reflected in the ABI “mental health” score.

**Supplementary Material**

**Table of Contents**

**Supplementary Figure 6.** Scatter plot of average latency of the first fixation on non-biological motion as a function of ASD severity level, as reflected in the ABC “stereotypic behavior” score.

**Supplementary Figure 7.** Scatter plot of average latency of the first fixation on non-biological motion as a function of ASD severity level, as reflected in the RBS-R “restricted behavior” score.

**Supplementary Figure 8.** Scatter plot of average latency of the first fixation on non-biological motion as a function of ASD severity level, as reflected in the RBS-R “stereotyped behavior” score.

**Effect of Stimulus Complexity on Attention to Non-Biological Motion Stimuli.**

**Effect of Familiarization with the Stimuli on Preference for Biological Motion.**

**Supplementary Table 1.** Results obtained with ANCOVA on different selections of participants’ data.

| Selection | | TD (n) | ASD (n) | Model parameters | | | Adjusted R^2^ |
| --- | --- | --- | --- | --- | --- | --- | --- |
| Eye movement metric | |  |  | Group | Gender | Age |  |
| Total valid time (%) | all data | 40 | 121 | **8.83 (<10^-3^)** | -1.65 (0.50) | 0.09 (0.45) | 0.07 |
|  | lib | 37 | 115 | **8.36 (<10^-4^)** | -0.99 (0.61) | 0.03 (0.70) | 0.10 |
|  | inter | 37 | 112 | **7.59 (<10^-4^)** | -1.49 (0.42) | 0.01 (0.89) | 0.10 |
|  | cons | 29 | 93 | **7.25 (<10^-4^)** | 1.68 (0.28) | 0.06 (0.40) | 0.13 |
| Preference for biological motion (%) | all data | 40 | 121 | **7.71 (<10^-5^)** | -0.16 (0.92) | **-0.17 (0.02)** | 0.14 |
|  | lib | 38 | 115 | **6.85 (<10^-6^)** | -0.83 (0.53) | **-0.13 (0.03)** | 0.16 |
|  | inter | 35 | 109 | **6.77 (<10^-7^)** | -0.75 (0.54) | -0.11 (0.06) | 0.19 |
|  | cons | 28 | 86 | **7.17 (<10^-10^)** | 0.36 (0.72) | -0.08 (0.08) | 0.32 |
| Time the first fixation was on biological motion (%) | all data | 40 | 121 | -1.25 (0.26) | 1.43 (0.20) | -0.05 (0.32) | 0.01 |
|  | lib | 38 | 116 | -1.85 (0.07) | 0.37 (0.72) | -0.07 (0.16) | 0.02 |
|  | inter | 37 | 102 | -1.20 (0.18) | 0.25 (0.78) | -0.05 (0.19) | 0.01 |
|  | cons | 30 | 87 | **-1.72 (0.03)** | 1.32 (0.08) | -0.04 (0.27) | 0.08 |
| Average latency of the first fixation on biological motion (msec) | all data | 40 | 121 | **-117.26 (0.01)** | **84.62 (0.05)** | -0.66 (0.73) | 0.07 |
|  | lib | 38 | 117 | **-112.27 (0.01)** | **69.72 (0.05)** | -0.75 (0.64) | 0.09 |
|  | inter | 35 | 112 | **-119.77 (<10^-3^)** | **77.61 (0.02)** | 0.21 (0.88) | 0.13 |
|  | cons | 29 | 88 | **-123.98 (<10^-5^)** | 27.47 (0.28) | -0.09 (0.94) | 0.18 |
| Average latency of the first fixation on non-biological motion (msec) | all data | 40 | 121 | **-105.52 (0.02)** | 4.87 (0.91) | -1.50 (0.46) | 0.02 |
|  | lib | 38 | 117 | **-132.54 (<10^-3^)** | -5.51 (0.88) | 0.27 (0.87) | 0.06 |
|  | inter | 37 | 107 | **-116.52 (<10^-3^)** | 34.98 (0.27) | 0.84 (0.55) | 0.09 |
|  | cons | 36 | 81 | **-156.38 (<10^-8^)** | -2.07 (0.94) | 1.32 (0.22) | 0.26 |

Data are shown as model parameter estimates along with the *P*-values in parentheses and adjusted coefficient of determination (R^2^). The *P*-values listed in parentheses are not adjusted for multiple comparisons. Positive values of the group and gender coefficients indicate greater eye movement metrics in the TD and male as compared to ASD and female participants, respectively, and vice versa. Potential outliers are filtered out by removing data points lying outside the range of population mean ± k × SD from models fitting. Several selection criteria are tested ranging from liberal (lib, k = 2) to conservative (cons, k = 1) including one intermediate level (inter, k = 1.5). The findings reported in the main text are based on ‘all data’ that denotes a combination of the two entire groups of participants. n indicates the number of participants. *P*-values < 0.05 are highlighted in bold. See Supplementary Figure 2 for a scatter plot of preference for biological motion as a function of participant’s age. See Supplementary Figure 3 for the distributions of average latency of the first fixation on biological motion in male and female participants.

ASD: autism spectrum disorder; cons: conservative; inter: intermediate; lib: liberal; SD: standard deviation; TD: typically developing.

**Supplementary Table 2.** Results obtained with ANCOVA using groups of the ASD participants with varying levels of intelligence quotient.

|  | KBIT-2 IQ composite score (range) | | TD (n) | ASD (n) | Model parameters | | | Adjusted R^2^ |
| --- | --- | --- | --- | --- | --- | --- | --- | --- |
| Eye movement metric |  |  |  |  | Group | Gender | Age |  |
| Total valid time (%) | | 60-136 | 40 | 121 | **8.83 (<10^-3^)** | -1.65 (0.50) | 0.09 (0.45) | 0.07 |
|  | | 60-84 | 40 | 34 | **15.26 (<10^-4^)** | 0.34 (0.93) | 0.08 (0.54) | 0.22 |
|  | | 85-115 | 40 | 63 | **6.93 (0.01)** | -1.73 (0.48) | 0.08 (0.49) | 0.09 |
|  | | 116-136 | 40 | 24 | 5.26 (0.06) | -2.95 (0.38) | -0.05 (0.70) | 0.03 |
| Preference for biological motion (%) | | 60-136 | 40 | 121 | **7.71 (<10^-5^)** | -0.16 (0.92) | **-0.17 (0.02)** | 0.14 |
|  | | 60-84 | 40 | 34 | 3.75 (0.07) | 0.12 (0.96) | -0.10 (0.25) | 0.03 |
|  | | 85-115 | 40 | 63 | **8.85 (<10^-6^)** | **-3.72 (0.05)** | **-0.22 (0.02)** | 0.24 |
|  | | 116-136 | 40 | 24 | **10.11 (<10^-3^)** | -1.05 (0.73) | -0.22 (0.07) | 0.21 |
| Time the first fixation was on biological motion (%) | | 60-136 | 40 | 121 | -1.25 (0.26) | 1.43 (0.20) | -0.05 (0.05) | 0.01 |
|  |  | 60-84 | 40 | 34 | **-3.00 (0.04)** | -0.004 (0.998) | -0.10 (0.10) | 0.06 |
|  | | 85-115 | 40 | 63 | -0.12 (0.93) | 1.60 (0.27) | -0.02 (0.81) | -0.01 |
|  | | 116-136 | 40 | 24 | -2.46 (0.10) | 0.51 (0.78) | -0.05 (0.46) | 0.02 |
| Average latency of the first fixation on biological motion (msec) | | 60-136 | 40 | 121 | **-117.26 (0.01)** | **84.62 (0.05)** | -0.66 (0.73) | 0.07 |
|  |  | 60-84 | 40 | 34 | **-204.78 (<10^-3^)** | 118.31 (0.07) | 0.17 (0.94) | 0.18 |
|  | | 85-115 | 40 | 63 | **-82.01 (0.03)** | 54.47 (0.19) | -3.42 (0.09) | 0.09 |
|  | | 116-136 | 40 | 24 | -55.76 (0.21) | **120.36 (0.03)** | -1.57 (0.47) | 0.13 |
| Average latency of the first fixation on non-biological motion (msec) | | 60-136 | 40 | 121 | **-105.52 (0.02)** | 4.87 (0.91) | -1.50 (0.46) | 0.02 |
|  |  | 60-84 | 40 | 34 | **-159.01 (0.02)** | 19.16 (0.78) | -2.45 (0.33) | 0.07 |
|  | | 85-115 | 40 | 63 | **-95.80 (0.04)** | 74.25 (0.15) | -2.07 (0.41) | 0.06 |
|  | | 116-136 | 40 | 24 | -32.04 (0.59) | 44.83 (0.53) | 0.03 (0.99) | -0.03 |

ANCOVA’s are conducted combining the data of all TD participants and individuals with ASD with a specific level of IQ. The *P*-values listed in parentheses are not adjusted for multiple comparisons. Each tested level is defined by the range of KBIT-2 IQ composite score. Same conventions and abbreviations as in Supplementary Table 1.

ASD: autism spectrum disorder; IQ: intelligence quotient; KBIT-2: Kaufmann Brief Intelligence Test-2; TD: typically developing.

**Supplementary Table 3.** Spearman correlations between the eye movement metrics and KBIT-2 IQ composite score in individuals with ASD.

|  |  | KBIT-2 IQ composite score | | | |
| --- | --- | --- | --- | --- | --- |
|  | range | 60-84 | 85-115 | 116-136 | 60-136 |
|  | mean (SD) | 73.0 (7.4) | 101.7 (8.3) | 126.2 (6.4) | 98.5 (20.0) |
| Eye movement metric | n | 34 | 63 | 24 | 121 |
| % Total valid time | | -0.077 (0.67) | **0.339 (<10^-2^)** | -0.137 (0.52) | **0.299 (<10^-3^)** |
| Preference for biological motion | | **0.409 (0.02)** | 0.014 (0.91) | -0.112 (0.60) | **-0.218 (0.02)** |
| % Time the first fixation was on biological motion | | 0.138 (0.44) | 0.083 (0.52) | -0.135 (0.53) | -0.015 (0.87) |
| Average latency of the first fixation on biological motion | | 0.031 (0.86) | **-0.254 (0.04)** | 0.115 (0.59) | **-0.232 (0.01)** |
| Average latency of the first fixation on non-biological motion | | 0.131 (0.46) | -0.005 (0.97) | 0.270 (0.20) | -0.141 (0.12) |

Data are shown as correlation coefficients along with the corresponding *P*-values in parentheses. Correlation coefficients are computed using the entire ASD sample as well smaller groups of individuals with ASD with a specific level of IQ. n indicates the number of participants. *P*-values < 0.05 are highlighted in bold.

ASD: autism spectrum disorder; KBIT-2: Kaufmann Brief Intelligence Test-2; SD: standard deviation.

**Supplementary Table 4.** Numeric scores of different ASD symptoms used to define three severity levels in Table 2.

|  | | Total range | Symptom severity level (mean [range]) | | |
| --- | --- | --- | --- | --- | --- |
|  | |  | Mild | Moderate | Severe |
| **ABI** |  |  | n = 39 | n = 39 | n = 39 |
| Core ASD symptom scale score |  | 0-6 | 1.14 [0.31-1.54] | 1.97 [1.56-2.31] | 2.95 [2.32-4.56] |
| Challenging behavior |  | 0-6 | 0.15 [0.00-0.50] | 0.88 [0.50-1.30] | 2.49 [1.33-5.30] |
| Mental health |  | 0-6 | 0.79 [0.00-1.18] | 1.65 [1.18-2.18] | 3.28 [2.27-5.00] |
| Restrictive repetitive behaviors |  | 0-6 | 1.05 [0.19-1.69] | 2.20 [1.69-2.88] | 3.70 [2.88-5.19] |
| Self-regulation |  | 0-6 | 0.83 [0.22-1.56] | 2.14 [1.56-2.78] | 4.07 [2.78-5.89] |
| Social communication |  | 0-6 | 1.03 [0.36-1.36] | 1.67 [1.39-2.00] | 2.76 [2.00-4.35] |
| **ADOS-2** | |  | n = 40 | n = 40 | n = 41 |
| Restricted and repetitive behavior | | 0-10 | 5.08 [1.00-7.00] | 8.18 [7.00-9.00] | 9.44 [9.00-10.0] |
| Social affect | | 0-10 | 5.28 [3.00-7.00] | 7.43 [7.00-8.00] | 9.32 [8.00-10.0] |
| Total score |  | 0-10 | 5.58 [4.00-7.00] | 7.75 [7.00-9.00] | 9.44 [9.00-10.0] |
| **ABC** |  |  | n = 40 | n = 40 | n = 41 |
| Hyperactivity non-compliance |  | 0-48 | 4.00 [0.00-8.00] | 13.83 [9.00-21.0] | 29.51 [21.0-43.0] |
| Inappropriate speech |  | 0-12 | 0.60 [0.00-2.00] | 3.20 [2.00-5.00] | 7.78 [5.00-12.0] |
| Irritability |  | 0-45 | 1.68 [0.00-4.00] | 7.68 [4.00-13.0] | 20.61 [13.0-38.0] |
| Lethargy social withdrawal |  | 0-48 | 2.93 [0.00-7.00] | 9.88 [7.00-13.0] | 20.93 [13.0-39.0] |
| Stereotypic behavior |  | 0-21 | 0.80 [0.00-2.00] | 3.83 [2.00-5.00] | 11.32 [5.00-21.0] |
| **CASI-Anx** | |  | n = 40 | n = 40 | n = 41 |
| Total score |  | 0-63 | 6.80 [0.00-11.0] | 14.65 [11.0-18.0] | 29.15 [19.0-54.0] |
| **RBS-R** | |  | n = 40 | n = 40 | n = 41 |
| Compulsive behavior | | 0-24 | 0.65 [0.00-2.00] | 3.10 [2.00-5.00] | 10.39 [5.00-22.0] |
| Ritualistic behavior | | 0-18 | 0.70 [0.00-2.00] | 4.25 [2.00-6.00] | 10.17 [6.00-17.0] |
| Restricted behavior | | 0-12 | 0.75 [0.00-2.00] | 3.13 [2.00-5.00] | 7.78 [5.00-12.0] |
| Sameness behavior | | 0-33 | 1.73 [0.00-4.00] | 6.65 [4.00-10.0] | 16.49 [10.0-30.0] |
| Self-injurious behavior | | 0-24 | 0.00 [0.00-0.00] | 1.08 [0.00-2.00] | 5.12 [2.00-19.0] |
| Stereotyped behavior | | 0-18 | 0.48 [0.00-2.00] | 3.43 [2.00-6.00] | 9.54 [6.00-18.0] |
| Total score |  | 0-129 | 8.10 [1.00-14.0] | 23.23 [15.0-34.0] | 54.22 [35.0-99.0] |
| **SRS-2** |  |  | n = 40 | n = 40 | n = 40 |
| Social awareness |  | 32-109 | 60.63[52.0-66.0] | 70.08 [66.0-76.0] | 85.23 [76.0-98.0] |
| Social cognition |  | 37-110 | 59.43[46.0-65.0] | 70.23 [65.0-76.0] | 84.35 [76.0-100] |
| Social communication |  | 37-115 | 62.38[44.0-68.0] | 73.53 [68.0-78.0] | 86.23 [78.0-102] |
| Social motivation |  | 37-110 | 55.30[42.0-62.0] | 66.65 [62.0-71.0] | 81.08 [71.0-104] |
| Restricted interests and repetitive  behavior | | 40-115 | 60.25[50.0-68.0] | 74.73 [68.0-82.0] | 90.43 [83.0-105] |
| Social communication and interaction | | 35-119 | 62.70[47.0-69.0] | 73.38 [70.0-78.0] | 86.33 [79.0-104] |
| Total score |  | 36-120 | 62.83[48.0-68.0] | 74.03 [69.0-79.0] | 88.05 [80.0-105] |

Data are shown as the mean numeric score along with its range for each examined ASD symptom and severity level. Participants with a score on the border between two contiguous severity levels were randomly assigned to one of the two levels to achieve a similar number of participants across levels.

ABC: Aberrant Behavior Checklist; ABI: Autism Behavior Inventory; ADOS-2: Autism Diagnostic Observation Schedule, 2^nd^ edition; CASI-Anx: Child Adolescent Symptom Inventory – Anxiety; RBS-R: Repetitive Behavior Scale – Revised; SRS-2: Social Responsiveness Scale 2™.

**Supplementary Table 5.** Spearman partial correlations between the eye movement metrics and the numeric scores of different ASD symptoms.

|  | |  |  | Eye movement metric | |  | |  | |  |
| --- | --- | --- | --- | --- | --- | --- | --- | --- | --- | --- |
|  | | % Total valid time | Preference for biological motion | % Time the first fixation was on biological motion | | Average latency of the first fixation on biological motion | | Average latency of the first fixation on non-biological motion | |  |
| ASD symptom | |  |  |  |  |  |  |  |  |  |
| **ABI (n = 117)** |  |  |  |  |  |  |  |  |  |  |
| Core ASD symptom scale score |  | -0.030 (0.75) | 0.117 (0.21) | 0.055 (0.56) | | 0.081 (0.39) | | -0.066 (0.48) | |  |
| Challenging behavior |  | -0.003 (0.98) | 0.160 (0.09) | -0.013 (0.89) | | -0.008 (0.94) | | -0.175 (0.06) | | |
| Mental health |  | 0.068 (0.47) | **0.185 (0.05)** | -0.098 (0.30) | | 0.050 (0.60) | | -0.091 (0.34) | | |
| Restrictive repetitive behaviors |  | -0.030 (0.75) | 0.181 (0.05) | 0.015 (0.88) | | 0.050 (0.60) | | -0.106 (0.26) | | |
| Self-regulation |  | -0.121 (0.20) | 0.081 (0.39) | 0.103 (0.27) | | 0.021 (0.83) | | -0.055 (0.56) | | |
| Social communication |  | 0.010 (0.92) | 0.021 (0.82) | 0.113 (0.23) | | 0.055 (0.56) | | -0.055 (0.56) | | |
| **ADOS-2 (n = 121)** | | | | | | | | | | |
| Restricted and repetitive behavior |  | **0.192 (0.04)** | 0.007 (0.94) | 0.171 (0.06) | | -0.098 (0.29) | | -0.037 (0.69) | | |
| Social affect |  | -0.037 (0.69) | -0.140 (0.13) | -0.117 (0.21) | | 0.033 (0.73) | | 0.067 (0.47) | | |
| Total score |  | 0.063 (0.50) | -0.118 (0.20) | -0.049 (0.60) | | -0.050 (0.59) | | 0.051 (0.58) | | |
| **ABC (n = 121)** | | | | | | | | | | |
| Hyperactivity non-compliance |  | -0.079 (0.40) | 0.036 (0.70) | 0.053 (0.57) | | -0.024 (0.79) | | -0.081 (0.38) | | |
| Inappropriate speech |  | -0.054 (0.56) | 0.093 (0.32) | 0.111 (0.23) | | -0.083 (0.37) | | -0.081 (0.38) | | |
| Irritability |  | 0.058 (0.53) | 0.158 (0.09) | -0.042 (0.65) | | -0.038 (0.68) | | -0.165 (0.07) | | |
| Lethargy social withdrawal |  | 0.128 (0.17) | -0.047 (0.61) | -0.009 (0.92) | | -0.023 (0.81) | | 0.020 (0.83) | | |
| Stereotypic behavior |  | -0.071 (0.45) | 0.148 (0.11) | 0.075 (0.42) | | -0.039 (0.67) | | **-0.245 (0.01)** | | |
| **CASI-Anx (n = 121)** | | | | | | | | | | |
| Total score |  | 0.026 (0.78) | 0.067 (0.47) | -0.029 (0.76) | | -0.052 (0.58) | | 0.006 (0.95) | | |
| **RBS-R (n = 121)** | | | | | | | | | | |
| Compulsive behavior |  | 0.154 (0.10) | 0.090 (0.33) | -0.057 (0.54) | | -0.065 (0.49) | | -0.090 (0.33) | | |
| Ritualistic behavior |  | 0.026 (0.78) | 0.147 (0.11) | -0.058 (0.53) | | 0.038 (0.68) | | -0.079 (0.39) | | |
| Restricted behavior |  | 0.141 (0.13) | 0.086 (0.35) | -0.035 (0.71) | | -0.121 (0.19) | | **-0.192 (0.04)** | | |
| Sameness behavior |  | 0.048 (0.61) | 0.150 (0.11) | 0.020 (0.83) | | -0.032 (0.73) | | -0.133 (0.15) | | |
| Self-injurious behavior |  | 0.047 (0.62) | 0.167 (0.07) | 0.095 (0.30) | | -0.058 (0.53) | | -0.024 (0.79) | | |
| Stereotyped behavior |  | -0.052 (0.57) | 0.093 (0.32) | 0.130 (0.16) | | -0.087 (0.35) | | **-0.191 (0.04)** | | |
| Total score |  | 0.047 (0.62) | 0.164 (0.08) | 0.005 (0.96) | | -0.036 (0.70) | | -0.138 (0.14) | | |
| **SRS-2 (n = 120)** | | | | | | | | | | |
| Social awareness |  | -0.052 (0.58) | 0.077 (0.41) | 0.084 (0.37) | | 0.001 (0.99) | | -0.123 (0.19) | | |
| Social cognition |  | 0.047 (0.62) | 0.088 (0.35) | 0.035 (0.71) | | -0.063 (0.50) | | -0.101 (0.28) | | |
| Social communication |  | 0.012 (0.90) | 0.071 (0.45) | -0.011 (0.91) | | 0.009 (0.92) | | -0.108 (0.25) | | |
| Social motivation |  | 0.103 (0.27) | 0.065 (0.49) | 0.021 (0.82) | | 0.013 (0.89) | | 0.007 (0.94) | | |
| Restricted interests and repetitive behavior |  | 0.024 (0.80) | 0.139 (0.13) | 0.001 (0.99) | | -0.012 (0.89) | | -0.153 (0.10) | | |
| Social communication and interaction |  | 0.031 (0.74) | 0.091 (0.33) | 0.005 (0.96) | | -0.012 (0.90) | | -0.101 (0.28) | | |
| Total score |  | 0.026 (0.78) | 0.112 (0.23) | 0.009 (0.93) | | -0.010 (0.91) | | -0.119 (0.20) | | |

Data are shown as correlation coefficients along with the corresponding *P*-values in parentheses. Correlation coefficients are computed on the data of all ASD participants with participant’s age, gender and KBIT-2 intelligence composite score being used as covariates. n indicates the number of participants. *P*-values < 0.05 are highlighted in bold.

ABC: Aberrant Behavior Checklist; ABI: Autism Behavior Inventory; ADOS-2: Autism Diagnostic Observation Schedule, 2^nd^ edition; CASI-Anx: Child Adolescent Symptom Inventory – Anxiety; KBIT-2: Kaufmann Brief Intelligence Test-2; RBS-R: Repetitive Behavior Scale – Revised; SRS-2: Social Responsiveness Scale 2™.

**Supplementary Table 6.** Comparison of variance of the eye movement metrics between the two groups of participants.

| Eye movement metric | Degrees of freedom | F-statistic | *P*-value |
| --- | --- | --- | --- |
| % Total valid time | (1, 159) | 10.02 | **0.002** |
| Preference for biological motion | (1, 159) | 2.56 | 0.11 |
| % Time the first fixation was on biological motion | (1, 159) | 0.21 | 0.65 |
| Average latency of the first fixation on biological motion | (1, 159) | 4.41 | **0.04** |
| Average latency of the first fixation on non-biological motion | (1, 159) | 3.66 | 0.06 |

The Levene’s test is used to assess the equality of variance of each eye movement metric between the two groups of participants. The tests are based on the data of all ASD (n = 121) and TD (n = 40) participants. *P*-values < 0.05 are highlighted in bold.

**Supplementary Table 7.** Results obtained with ANCOVA when correcting for the violations of homogeneity of variance.

| Eye movement metric | df | Model parameters | | | | | |
| --- | --- | --- | --- | --- | --- | --- | --- |
|  |  | Group | | Gender | | Age | |
|  |  | F-statistic | *P*-value | F-statistic | *P*-value | F-statistic | *P*-value |
| % Total valid time | (1, 157) | 21.33 | **< 10^-5^** | 0.45 | 0.50 | 0.92 | 0.34 |
| Preference for biological motion | (1, 157) | 19.97 | **< 10^-4^** | 0.01 | 0.93 | 6.40 | **0.02** |
| % Time the first fixation was on biological motion | (1, 157) | 1.13 | 0.29 | 1.53 | 0.22 | 1.71 | 0.19 |
| Average latency of the first fixation on biological motion | (1, 157) | 11.63 | **< 10^-3^** | 4.22 | **0.05** | 0.10 | 0.75 |
| Average latency of the first fixation on non-biological motion | (1, 157) | 5.33 | **0.03** | 0.01 | 0.91 | 0.66 | 0.42 |

ANCOVA’s are conducted combining the data of all TD participants and individuals with ASD (see Selection “all data” in Supplementary Table 1). In each ANCOVA, the HC3 correction is used to account for the violations of homogeneity of variance. *P*-values are not adjusted for multiple comparisons, with the values below 0.05 being highlighted in bold.

df: Degrees of freedom.

**Supplementary Figure 1.** Biological (left) and non-biological (right) motions with corresponding ROIs.


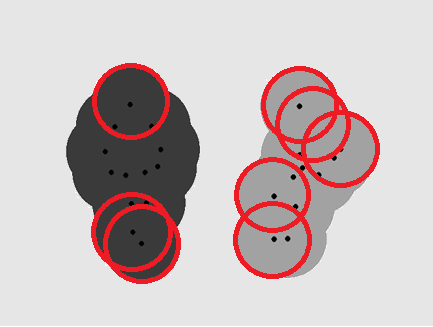


Each of the two ROIs is made up of circles (in red) with the radius of approximately 3.7° of visual angle centered at each “black dot” that correspond to a (non-)biological motion stimulus. For the sake of clarity, only a portion of all “red circles” is shown. Note that the unions of circles denoted by shaded areas (biological motion – in dark grey color, non-biological motion – in light grey color) were invisible to participants during actual experiments and are presented here for the sake of illustration only.

ROI: Regions of interest.

**Supplementary Figure 2.** Scatter plot of preference for biological motion as a function of participant’s age.


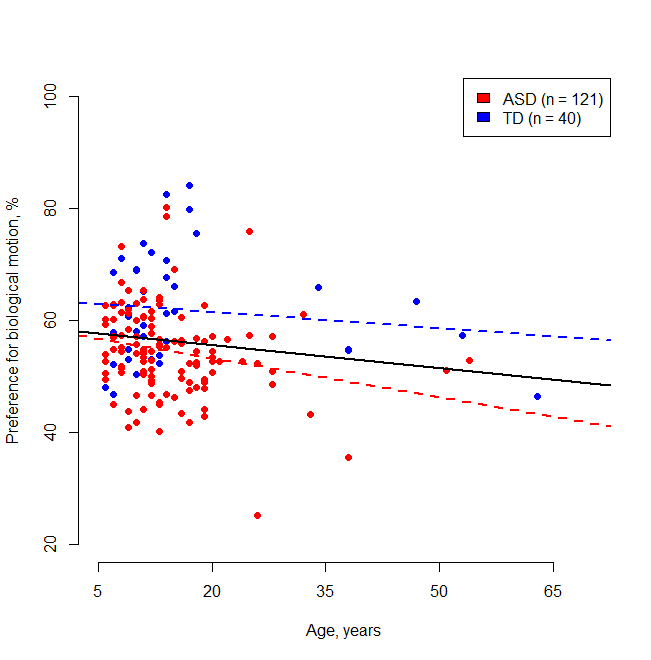


Data points denote individual participants. The red and blue colors correspond to the data of ASD (Spearman correlation coefficient r_S_ = -0.259, *P* < 0.01) and TD (r_S_ = 0.211, *P* = 0.19) groups, respectively. n indicates the number of participants in each group. The dashed lines correspond to the best linear fits of the data of each participant group separately. The solid black line is the best linear fit obtained when the data of both groups are combined (r_S_ = -0.161, *P* < 0.05).

ASD: autism spectrum disorder; TD: typically developing.

**Supplementary Figure 3.** Histograms of average latency of the first fixation on biological motion for each participant’s gender separately.


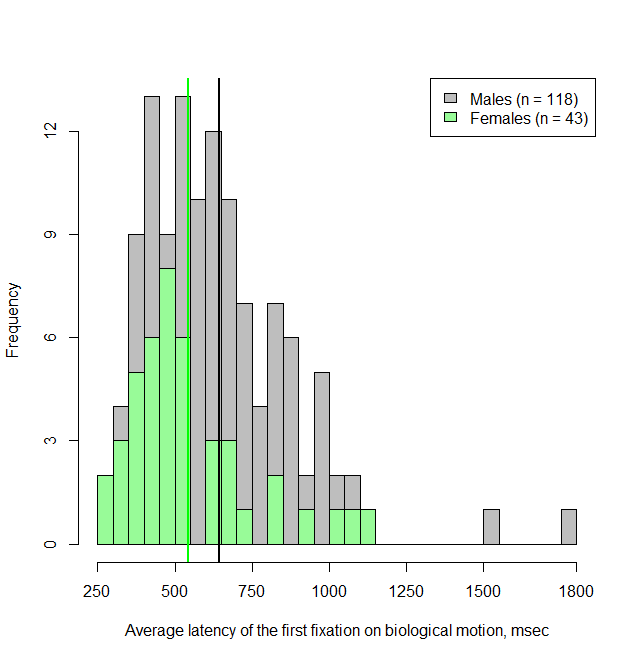


Data are pooled across both individuals with ASD and TD controls. The gray and pale green colors correspond to the data of male and female participants, respectively. The solid vertical black and light green lines denote the mean values of average latency of the first fixation on biological motion in male and female participants, respectively. n indicates the number of participants. Bin width is 50 msec.

ASD: autism spectrum disorder; TD: typically developing

**Supplementary Figure 4.** Scatter plot of % total valid time as a function of ASD severity level, as reflected in the ADOS “restricted and repetitive behavior” score.


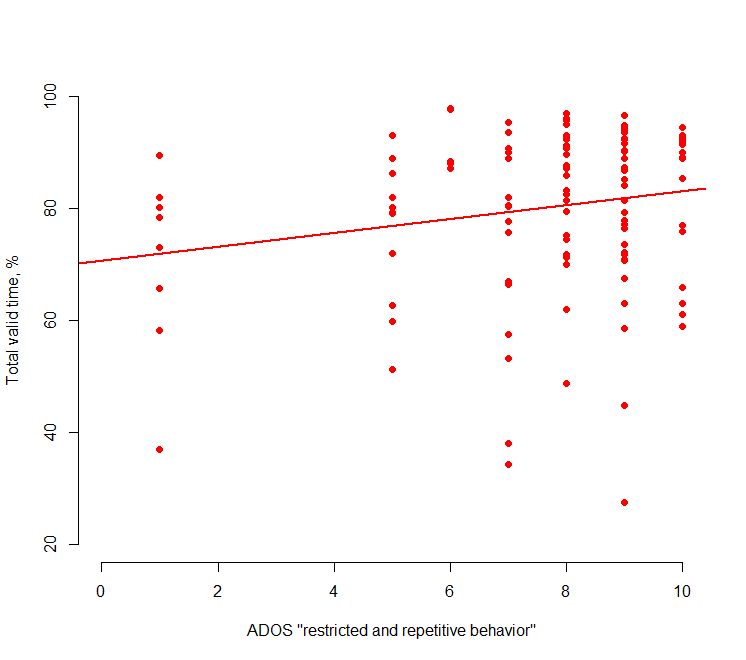


Data points denote individuals with ASD (n = 121). The red line corresponds to the best linear fit of the presented data (Spearman partial correlation coefficient r_S_ = 0.192, *P* < 0.04; see Supplementary Table 5).

ADOS: Autism Diagnostic Observation Schedule, 2^nd^ edition; ASD: autism spectrum disorder.

**Supplementary Figure 5.** Scatter plot of preference for biological motion as a function of ASD severity level, as reflected in the ABI “mental health” score.


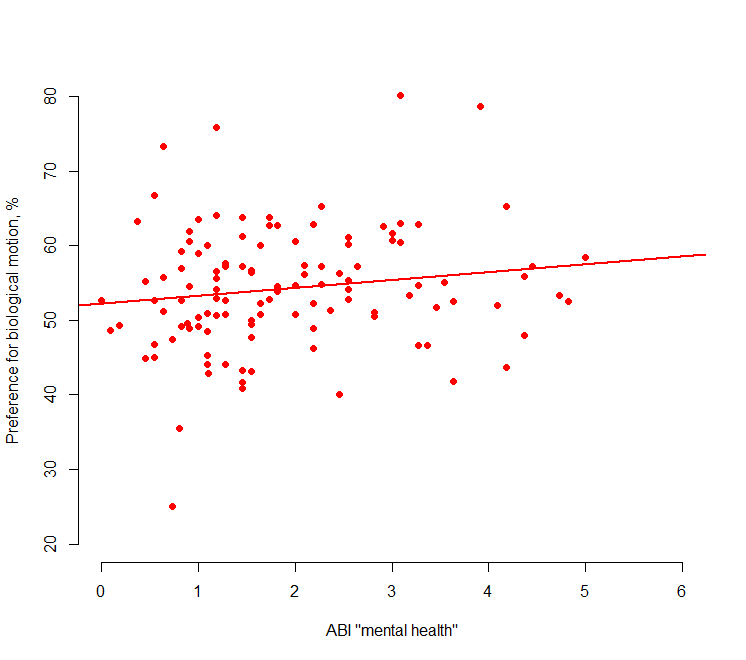


Data points denote individuals with ASD (n = 121). The red line corresponds to the best linear fit of the presented data (Spearman partial correlation coefficient r_S_ = 0.185, *P* < 0.05; see Supplementary Table 5).

ABI: Autism Behavior Inventory; ASD: autism spectrum disorder.

**Supplementary Figure 6.** Scatter plot of average latency of the first fixation on non-biological motion as a function of ASD severity level, as reflected in the ABC “stereotypic behavior” score.


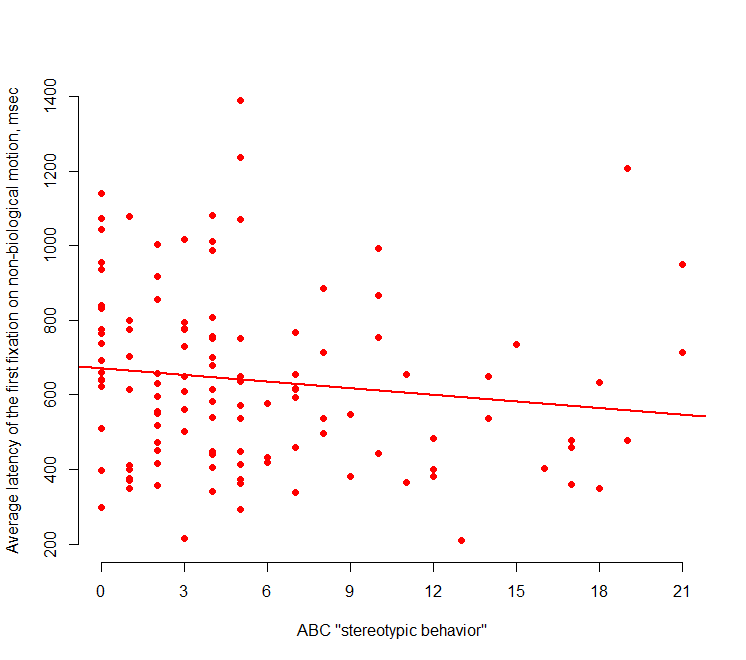


Data points denote individuals with ASD (n = 121). The red line corresponds to the best linear fit of the presented data (Spearman partial correlation coefficient r_S_ = -0.245, *P* < 0.01; see Supplementary Table 5).

ABC: Aberrant Behavior Checklist – Community; ASD: autism spectrum disorder.

**Supplementary Figure 7.** Scatter plot of average latency of the first fixation on non-biological motion as a function of ASD severity level, as reflected in the RBS-R “restricted behavior” score.


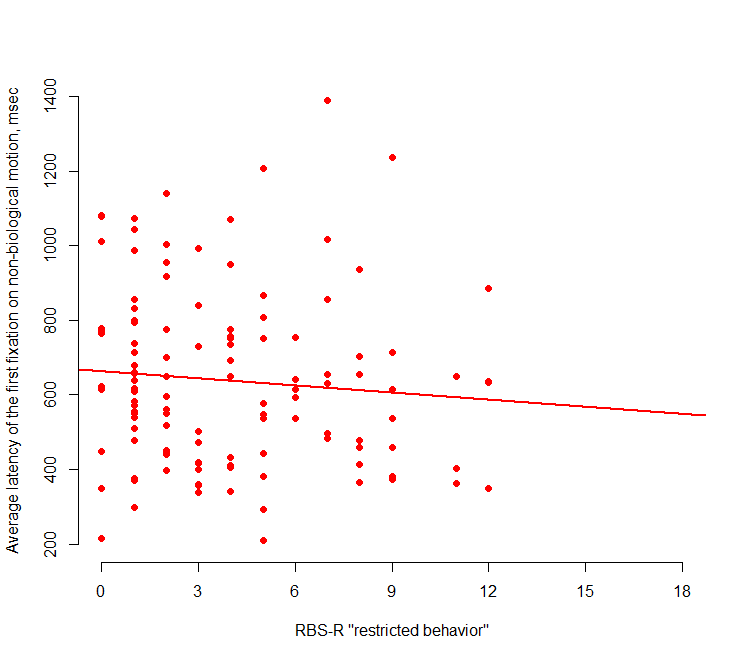


Data points denote individuals with ASD (n = 121). The red line corresponds to the best linear fit of the presented data (Spearman partial correlation coefficient r_S_ = -0.192, *P* < 0.04; see Supplementary Table 5).

ASD: autism spectrum disorder; RBS-R: Repetitive Behavior Scale – Revised.

**Supplementary Figure 8.** Scatter plot of average latency of the first fixation on non-biological motion as a function of ASD severity level, as reflected in the RBS-R “stereotyped behavior” score.


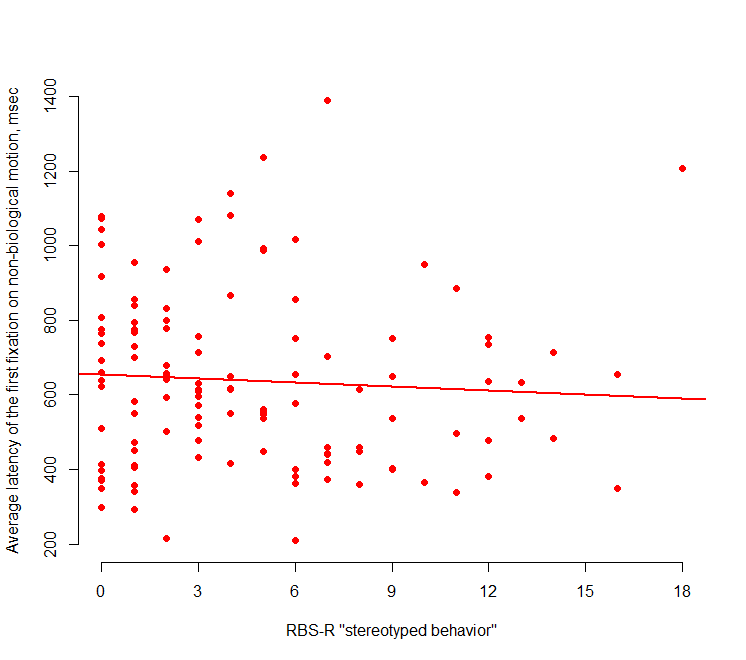


Data points denote individuals with ASD (n = 121). The red line corresponds to the best linear fit of the presented data (Spearman partial correlation coefficient r_S_ = -0.191, *P* < 0.04; see Supplementary Table 5).

ASD: autism spectrum disorder; RBS-R: Repetitive Behavior Scale – Revised.

**Effect of Stimulus Complexity on Attention to Non-Biological Motion Stimuli**

Non-biological motion was represented by two types of stimuli. These were either phase-scrambled versions of the biological motion (i.e. random movements of the dots in point-light displays) or continuous rotation of the first frame (i.e. spinning around the vertical axis) of the biological motion animation. Modelling preference for non-biological motion using linear mixed-effect model with random intercept and participant’s age, gender, type of non-biological stimuli, group, and interaction between the latter two factors as fixed effects revealed a greater attention to random movements of the dots than to their rotation, and this was true in both groups of participants (*P*-value < 10^-5^). Importantly, no interaction between participant group and type of non-biological motion stimuli was observed (*P*-value = 0.46). This suggests a similar preference for the two types of non-biological motion stimuli in both groups of participants. The latter rules out an explanation of lower preference for biological motion in individuals with ASD as compared to TD controls by differences in attention to non-biological motion stimuli between the two groups of participants.

**Effect of Familiarization with the Stimuli on Preference for Biological Motion**

To test whether preference for biological motion was strengthened over repeated trials of stimuli (i.e. familiarization with the stimuli), preference for biological motion was modelled using a linear mixed-effect model for repeated measures. The model included participant group (TD vs. ASD), (non-)biological motion stimulation duration and their interaction as covariates/factors. Three different stimulation durations were analysed. These included (1) first 1/6 of the entire stimulation duration of (non-)biological motion stimuli, (2) first 1/3, and (3) the entire duration of the biological motion test. As a result, there was no effect of stimulation duration on preference for biological motion found (*P*-value = 0.90). Moreover, there was no significant interaction between participant group and stimulation duration (*P*-value = 0.52). Altogether, this rules out an explanation of the difference in preference for biological motion and its modulation between the two groups of participants by stimulation duration. Importantly, the model revealed a significant difference in preference for biological motion between the two groups of participants (*P*-value < 0.0001) that agrees with the reported findings.
